# Gamma estimator of Jarzynski equality for recovering binding energies from noisy dynamic data sets

**DOI:** 10.1038/s41467-020-19233-7

**Published:** 2020-11-02

**Authors:** Zhifeng Kuang, Kristi M. Singh, Daniel J. Oliver, Patrick B. Dennis, Carole C. Perry, Rajesh R. Naik

**Affiliations:** 1 Air Force Research Laboratory, Wright-Patterson Air Force Base, OH, 45433 USA; 2 Biomolecular and Materials Interface Research Group, Interdisciplinary Biomedical Research Centre, School of Science and Technology, Nottingham Trent University, Clifton Lane, Nottingham, NG11 8NS UK

**Keywords:** Protein design, Nanoscale biophysics, Single-molecule biophysics, Computational biology and bioinformatics, Computational models

## Abstract

A fundamental problem in thermodynamics is the recovery of macroscopic equilibrated interaction energies from experimentally measured single-molecular interactions. The Jarzynski equality forms a theoretical basis in recovering the free energy difference between two states from exponentially averaged work performed to switch the states. In practice, the exponentially averaged work value is estimated as the mean of finite samples. Numerical simulations have shown that samples having thousands of measurements are not large enough for the mean to converge when the fluctuation of external work is above 4 *k*_B_*T*, which is easily observable in biomolecular interactions. We report the first example of a statistical gamma work distribution applied to single molecule pulling experiments. The Gibbs free energy of surface adsorption can be accurately evaluated even for a small sample size. The values obtained are comparable to those derived from multi-parametric surface plasmon resonance measurements and molecular dynamics simulations.

## Abstract (web summary)

Measuring interaction energies from experimentally measured single-molecular interactions is challenging. Here, the authors report a gamma work distribution applied to single molecule pulling events for estimating peptide absorption free energy.

## Introduction

Protein adsorption on surfaces is a common yet complicated phenomenon^1,2^. Understanding the adsorption mechanism is essential in developing new approaches and strategies for biomedical implants, drug encapsulation and delivery, biotemplated material synthesis, antifouling, biosensing, and many other applications. The reconstruction of adsorption free energy profiles along a reaction coordinate offers insights into the thermodynamic and kinetic properties of an adsorption process.

A number of experimental techniques have been developed to determine adsorption free energies of proteins. Multi-parametric surface plasmon resonance spectroscopy (MP-SPR) and adsorption measurements using a quartz crystal microbalance (QCM) are two commonly used label-free techniques to quantify protein adsorption energies on surfaces. These techniques monitor real-time adsorption and desorption processes of analyte flowing through a channel and binding to a target. Kinetic information can be used to extract adsorption free energy by fitting a theoretical model, such as the Langmuir model^3–5^. The theoretical basis for this type of measurement is the chemical potential equality at equilibrium. According to thermodynamic law, the adsorption free energy is estimated as ${\Delta} G = k_{\mathrm{B}}T\ln \frac{{K_{\mathrm{D}}}}{{c^ \ominus }}$, where *K*_D_ is the dissociation equilibrium constant, *k*_B_ is the Boltzmann constant, *T* is the temperature in Kelvin, and *c*^⊖^ is the standard reference concentration (1 M). A long-standing problem is to recover the macroscopically observed Δ*G* from experimentally measured single-molecule interactions^6,7^.

The Jarzynski equality (JE) opens up the possibility of extracting adsorption free energy from single-molecule pulling experiments (SMPE) using atomic force microscopy^6–9^. The JE relates the adsorption free energy Δ*G* to the ensemble average of the Boltzmann weights of the external work *W* required in moving the protein from an adsorbed state to a free state in bulk solution:

1 $$e^{ - \beta {\Delta} G} = \left\langle {e^{ - \beta W}} \right\rangle$$

where *β* = 1/*k*_B_*T* and the bracket is the ensemble average of the Boltzmann weights of the external work *W* over an infinite number of repeated experiments. In the following, we will use *k*_B_*T* as the units of work and energy so the symbol *β* is dropped.

The JE forms a rigorous theoretical basis for the extraction of equilibrium free energy change from non-equilibrium single-molecule pulling experiments. The commonly used JE estimator $\widehat {{\Delta} G}_{{\mathrm{MN}}}$ is the mean of finite exponentially weighted work values from experiments:

2 $$\widehat {{\Delta} G}_{{\mathrm{MN}}} = - \log \left( {\frac{1}{N}\mathop {\sum}\limits_{i = 1}^N {e^{-W_i}} } \right)$$

However, $\widehat {{\Delta} G}_{{\mathrm{MN}}}$ estimates are notoriously difficult to converge. It has been shown that when the standard deviation (SD) of work is above 4 *k*_B_*T*, a sample size as large as 500,000 is not large enough for the ${\Delta} G_{{\mathrm{MN}}}$ to converge^10,11^. The $\widehat {{\Delta} G}_{{\mathrm{MN}}}$ estimates are strongly biased by small work values that rarely happen. The trajectories corresponding to the small work values may not appear in limited pulling repetitions or may be ignored by operators. This leads to unreproducible results and discrepancies in reported results^12–14^.

To overcome the difficulty, correction and extrapolation schemes have been proposed in post-processing^15,16^. Methods to limit large perturbations in the first place have also been proposed^6,9,17^. Among them, the stiff-spring approximation has proved efficient^18^. Under the stiff-spring approximation, the work distribution is expected to be Gaussian with a small deviation such that the average of exponential work can be reduced to the fluctuation-dissipation (FD) theorem ${\Delta} G = \left\langle W \right\rangle - \frac{1}{2}\sigma ^2$, where *σ* is the standard deviation of the work distribution. Thus the free energy can be estimated without an exponential operation as^18^:

3 $$\widehat {{\Delta} G}_{{\mathrm{FD}}} = \hat W - \frac{1}{2}\widehat {\sigma ^2}$$

where $$\hat W = \frac{1}{N}\mathop {\sum}\limits_{i = 1}^N {W_i}$$ and $$\widehat {\sigma ^2} = \frac{N}{{N - 1}}\left[ {\frac{1}{N}\mathop {\sum}\limits_{i = 1}^N {W_i^2} - \left( {\frac{1}{N}\mathop {\sum}\limits_{i = 1}^N {W_i} } \right)^2} \right]$$.

The FD theorem is valid when the fluctuation of work is comparable to temperature^6,18^. We refer to Eqs. (2) and (3) as the mean and FD estimator of the JE, respectively.

However, in protein adsorption studies using SMPE, it is easily observed that the work fluctuation is large, ranging from a few *k*_B_*T* to hundreds. Even for a 12-mer short peptide A3 (Ala-Tyr-Ser-Ser-Gly-Ala-Pro-Pro-Met-Pro-Pro-Phe)^19^, the reported adsorption free energies show large differences. The A3 peptide is a well-studied gold binding peptide previously identified from a combinatorial phage display peptide library. Heinz et al. proposed that the entropic gain due to interfacial water release compensates the entropic loss due to peptide binding on a surface so that the calculated enthalpy change −106 *k*_B_*T* is an acceptable approximation of binding free energy^20^. Tang et al. argued that the entropic contribution due to a large number of adsorbed conformations must be accounted for to interpret a QCM measured binding adsorption free energy −12.8 *k*_B_*T*^4^. To investigate the discrepancy, we have studied the binding of peptide A3 on a gold (111) surface using SMPE. Indeed, we have found the above two estimators of JE are not applicable. For the mean estimator $\widehat {{\Delta} G}_{{\mathrm{MN}}}$, the results differ from one experiment to another due to the available small sample sizes of work values obtained from experiments. Since the work fluctuation is large, the Gaussian work distribution estimator $\widehat {{\Delta} G}_{{\mathrm{FD}}}$ is not applicable either. Motivated by recent theoretical work^11^, we have observed that the underlying work distribution is a gamma distribution. For a given gamma distribution with probability density function

4 $$\rho \left( W \right) = \lambda ^\alpha W^{\alpha - 1}e^{ - \lambda W}{\mathrm{/}}{\Gamma} \left( \alpha \right)\,{\mathrm{for}}\,W \,> \, 0,$$

an exact expression for Δ*G* can be obtained as^11^

5 $${\Delta} G_{{\mathrm{GA}}} = \alpha \ln \left( {\frac{{\lambda + 1}}{\lambda }} \right)$$

where *α* and *λ* are the shape and rate parameters of the gamma distribution. For a finite set of experimental data $\left\{ {W_i} \right\}_{i = 1}^N$, the parameters *α* and *λ* can be estimated by maximizing the log of the likelihood function as

6 $$L = \mathop {\sum}\limits_{i = 1}^N {\log } (\lambda ^\alpha W_i^{\alpha - 1}e^{ - \lambda W_i}{\mathrm{/}}{\Gamma} \left( \alpha \right))$$

We refer to $\widehat {{\Delta} G}_{{\mathrm{GA}}}$ as the gamma estimator of JE. To implement the gamma estimator for peptide adsorption free energy calculations, pulling trajectories for single-peptide binding events must first be sorted out. Such a task is difficult to manually accomplish because real force-distance curves exhibit complicated patterns^12^. Large efforts have been devoted to both automatically and reliably sorting out single binding events^21–29^. To the best of our knowledge, there is no open-source code available for free energy calculations. We have developed the R package *afmFreeEnergy* tool; the implementation code is freely available at https://github.com/comtook/afmFreeEnergy. The efficiency of the procedure has been demonstrated by calculating the A3 peptide adsorption free energy on a gold surface. Furthermore, the estimated adsorption free energy is compared with that obtained from MP-SPR measurements and molecular dynamics simulation using the adaptive biasing force (ABF) algorithm^30–33^.

## Results

### Single-peptide pulling experiments

**Fig. 1:**
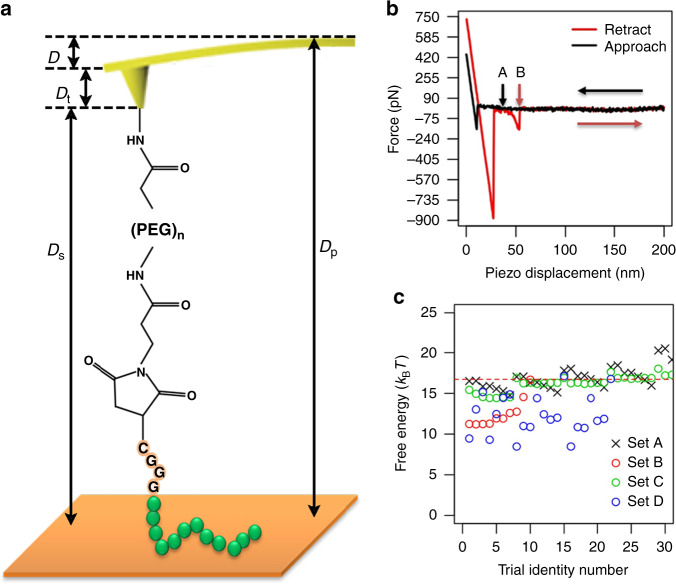
Free energy estimates from atomic force microscopy (AFM) pulling experiments. **a** Schematic of the AFM tip modification and experimental setup; *D*_p_ is the piezo displacement; *D*_s_ is the tip-sample separation distance; *D*_t_ is the height of the tip; *D* is the deflection. **b** Representative force-distance curve consisting of two segments: approach to surface (black horizontal arrow) and retraction from surface (red horizontal arrow); the vertical black and red arrows correspond to the two quasi-equilibrium states A (bound peptide) and B (free peptide). **c** The different combinations of experimental parameters yield different free energy values and converge to a unique value, as indicated by the dashed red line.

A schematic representation of the AFM experimental setup is shown in Fig. 1a. The distance *D*_s_ from the tip to the surface is called the separation distance. The distance *D*_p_ from the cantilever to surface is called the piezo displacement. The AFM tip is functionalized with the peptide via a heterobifunctional maleimide (MAL) terminated polyethylene glycol (–PEG–)_n_ linker. The linker serves two functions—to extend the peptide away from the tip and to provide rotational freedom for the peptide to interact with the surface. To facilitate coupling of the A3 peptide to the linker, a tetrapeptide segment containing a cysteine residue and three glycine residues are appended on the C-terminus of the A3 peptide (AYSSGAPPMPPF-GGGC).

When the cantilever moves towards (approach) and away (retraction) from the gold surface, it will deflect from its horizontal level due to interfacial interactions between the surface and molecular entities on the tip. The cantilever deflection (distance) can be detected and converted into interaction force using Hooke’s law and the force-distance curves are acquired. A representative force-distance curve (FDC) is shown in Fig. 1b.

When the tip is far away from the surface, it experiences no interaction with the surface so that the force in this bulk region fluctuates around zero according to a Gaussian distribution due to molecular thermal motion. We have used a linear regression method to align the force-distance curve so that the force is around zero when the tip is away from the surface. We have used a parameter *Alpha* = *0.5* ~ *0.7* to indicate the percent of collected data points that are away from the surface. A true interaction force signal must be above a certain noise level *LOD* * *σ*, where *σ* is the standard deviation of the Gaussian noise, and *LOD* is the limit of detection.

When the tip is close to the surface, van der Waals and electrostatic repulsive interactions push the cantilever to deflect upward, where the resulting force is positive. When the repulsive force reaches a threshold, the tip is paused for seconds to allow the peptide to fully relax on the surface. Then the cantilever is retracted away from the surface at a constant pulling rate. The first negative force minimum close to *D*_p_ = 0 is mainly due to van der Waals and electrostatic attractive interactions between the tip and the surface. When the cantilever moves to a point indicated by the black vertical arrow in Fig. 1b, the PEG linker is fully relaxed, whereas the peptide remains in contact with the surface. We define the state corresponding to this point as the peptide binding state A. Starting from this point, a parabolic segment representing the stretching of the PEG linker is observed. When the peptide is completely pulled away from the surface, the force abruptly returns to zero, which is observed as a step function shape in the FDC. We define this state as the free state B as indicated with the vertical red arrow in Fig. 1b. The integration from A to B along the parabolic segment is the required external work to pull the peptide from its binding state (state A) to its free state (state B). This is the work value used in the JE equation.

### Data processing

As commonly seen in AFM measurements, most of the collected curves do not meet the features described above. Thus, it is highly desirable to quantify selected features to automatically screen all collected curves for those satisfying the above-described features. To this end, we have developed an R package named *afmFreeEnergy* to automatically search for those curves satisfying the above-described features. First, we use a linear regression method to align the force-distance curve so that the force is around zero in the non-interaction bulk region. Second, we locate the state B noticing that it corresponds to a discontinuous point in the force-distance curve. This implies that it corresponds to a maximum of the derivative of the force-distance curve. Third, the minimum negative force close to state B must be less than −*LOD* * *σ*, representing a true binding signal. Fourth, we locate A having minimum absolute force in the baseline bounded by *mulBase* * *σ* closest to B, where *mulBase* = 2 ~ 4, depending on perturbation level. Lastly, the parabolic segment is fitted into the worm-like chain (WLC) model. For each curve meeting the above criteria, we calculate the work value by integrating the parabolic segment from point A to point B. Using the obtained work, the free energy difference between State A and State B are estimated.

### Free energy calculations

The free energy difference between states A and B is independent of the trajectory pathways and pulling rates, so a hard criterion for verification of the calculated free energy difference is that the obtained values must be the same, to within experimental error, from different pulling experiments at different pulling rates. To meet this criterion, we have collected two sets of data at two different pulling rates 500 nm/s and 1000 nm/s. To ensure whether the results are reproducible, we have repeated the process at different surface contact areas at the pulling rate 500 nm/s. We also obtained additional data from the A3 peptide without the C-terminus GGG linker. We acquired and processed four data sets A, B, C and D at the pulling rates 500 nm/s, 1000 nm/s, 500 nm/s, and 500 nm/s, respectively. The raw data consists of 812, 1667, 501, and 785 curves, respectively, and are shown in Supplementary Fig. 1a–d with details of the experimental parameters listed in Supplementary Table 1.

Using *afmFreeEnergy*, we automatically sorted curves meeting the established criteria based on four parameters *Alpha*, *LOD*, *mulBase*, and *nlsEr*. *mulBase* is the baseline fluctuation level allowing baseline force fluctuation bounded by *mulBase* * *σ*. *nlsEr* is the error tolerance bounded by *nlsEr* * *σ* for WLC fitting average residues. Setting *Alpha* = 0.5 *or* 0.7, *mulBase* = 2 *or* 3, *nlsEr* = 2 *or* 3, and varying *LOD* from 3 to 9, we have obtained different free energy estimates using the three estimators. However, the gamma estimates converge to a unique value 16.77 ± 0.07 *k*_B_*T* as shown in Fig. 1c, indicated by the red dashed line. The parameters, estimates and test statistics close to this unique value for each dataset are listed in Table 1. The selected curves based on the parameters in Table 1 are shown in Supplementary Fig. 1e–h.

**Table 1:** Parameters and statistics for A3-gold adsorption free energy estimates.

Dataset		A	B	C	D
parameters	*Alpha*	0.5	0.5	0.5	0.7
	*mulBase*	3	2.4	2.6	4
	*LOD*	5.5	5.5	5.5	9
	*nlsEr*	3	2	3	3
Mean	Samples	171	48	79	47
	*ΔG*_MN_	49.46	47.85	42.92	85.12
Gamma	*ΔG*_GA_	16.67	16.84	16.79	16.78
	Shape	4.198	4.42	5.11	3.968
	Rate	0.019	0.023	0.039	0.015
	*D*_KS_	0.037	0.069	0.059	0.086
	*P*	0.976	0.96	0.93	0.853
Norm	*ΔG*_FD_	−5140	−4431	−1532	−8619
	Mean	219	195	132	268
	Sd	104	96	58	133
	*D*_KS_	0.069	0.11	0.091	0.113
	*P*	0.397	0.62	0.49	0.546
	Prob	0.017	0.02	0.011	0.022

*D*_KS_ is the Kolmogorov–Smirnov test statistic; *P* is the *P*-value representing the likelihood of observing the test statistic; Prob is the probability of taking a negative work value.

**Fig. 2:**
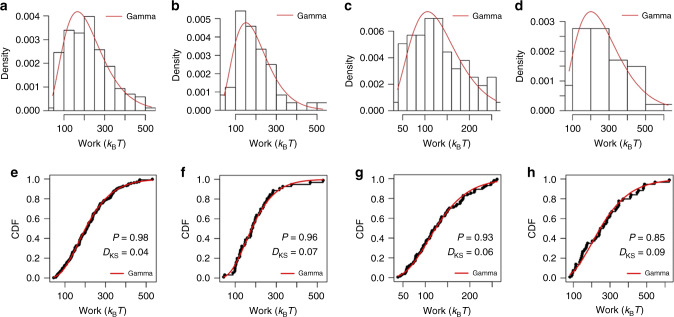
The statistics of gamma fitting. **a**–**d** Histograms of work values fitted with gamma distribution for datasets A, B, C, and D, respectively. **e**–**h** The empirical cumulative distribution function (CDF) comparing to theoretical gamma distribution for datasets A, B, C, and D, respectively. *D*_KS_ is the Kolmogorov–Smirnov test statistic. *P* is the P-value representing the likelihood of observing the test statistic.

Using the identified curves shown in Supplementary Fig. 1e–h, the external work values are calculated. The histograms of the work values with gamma distribution fits are shown in Fig. 2a–d for dataset A, B, C, and D, respectively. The empirical cumulative distribution function $\frac{{i - 0.5}}{N},i = 1, \cdots N$ (*N* is the number of data points) comparing to the calculated gamma distribution using Eq. (4) is shown in Fig. 2e–h.

The statistical hypothesis that the work values may be represented by a gamma distribution is tested using the Kolmogorov–Smirnov test^34^. The *P*-values for data sets A, B, C, and D are 0.98, 0.96, 0.93, and 0.85, respectively, which strongly support the hypothesis. The gamma estimator of JE produces the estimate of the free energy 16.67, 16.84, 16.79, and 16.78 *k*_B_*T* for data sets A, B, C, and D, respectively.

The mean estimator produces the free energy 49.46, 47.85, 42.92, and 85.12 *k*_B_*T* for datasets A, B, C, and D, respectively. Thus, different datasets yielded different values, and no convergent result was obtained. This clearly implies that, based on small experimental sample sizes, caution must be exercised in estimating the free energy using the mean JE estimator.

If the work distribution is modeled by a Gaussian distribution as shown in Supplementary Fig. 2, the probability of having negative work values for dataset A, B, C, and D is 0.017, 0.02, 0.011, and 0.022 because of large fluctuations *σ* = 104, 96, 58, and 133 *k*_B_*T*, respectively. In this case, the FD estimators are not applicable, since in our experimental setting negative work values should never occur. This implies that the negative work values in the left tail of a Gaussian distribution overestimate and dominate the free energy estimates, which yield nonphysical results −5140, −4431, −1532, and −8619 *k*_B_*T*. Only the gamma estimator gives consistent results in all four cases.

First, note that the gamma density curves in Fig. 2a–d are drawn using Eq. (4) with parameters *α* = (4.198, 4.42, 5.11, 3.968) and *λ* = (0.019, 0.023, 0.039, 0.015) which maximize the likelihood function (Eq. (6)) for dataset A, B, C, and D, respectively. The histograms under the curves are added for comparison and the bin sizes have no impact on the free energy estimates, as shown in Supplementary Fig. 3. Assuming the data come from a gamma distribution, we have found that the maximum likelihood method is consistent and efficient for estimating the parameters characterizing the distribution. It would be very appealing if we could nonparametrically learn a distribution model directly from data by plotting a histogram. We provide a description in deriving a distribution model from a histogram in Supplementary Note 1.

**Fig. 3:**
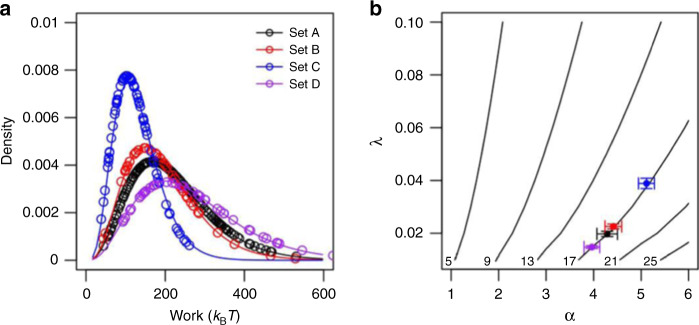
Gamma distribution and adsorption free energy estimates. **a** Curves represent the gamma fitting derived from total work data; circles represent the gamma fitting derived from the average of 10X random selection of 90% of total work data. The agreement shows that there is no overfitting. The gamma distribution shapes for datasets A, B, C, and D are different, but they converge to the same small work value ~17 *k*_B_*T* which is crucial for adsorption free energy estimates. **b** The solid contour lines show that the free energy 5, 9, 13, 17, 21, and 25 *k*_B_*T* can be achieved by infinite combination of shape and rate parameter α and λ of a gamma distribution. The estimates for datasets A, B, C, and D coincide with the contour line of 17*k*_B_*T*, and thus the estimated adsorption free energies agree well with the theoretical value ~17 *k*_B_*T*.

Second, to investigate whether there is overfitting, we have randomly shuffled the obtained work values and randomly selected 90% to repeat the gamma fitting (10 times). The obtained average gamma distribution parameters *α* = (4.284 ± 0.215, 4.414 ± 0.174, 5.113 ± 0.169, 3.964 ± 0.165) and *λ* = (0.0197 ± 0.000996, 0.0226 ± 0.000923, 0.0389 ± 0.00196, 0.0148 ± 0.000544) for dataset A, B, C, and D agree very well with the point estimates of the full work values. Figure 3a shows the gamma density functions derived from total or partial data sets agree well for each dataset. This implies that there is no overfitting.

Third, we noticed the shape of the work distribution of dataset C is different from the others. The cumulative distribution function for dataset C converges towards 1.0 about 250 *k*_B_*T* while others accumulate towards 1.0 about 600 *k*_B_*T*. Since work values depend on peptide binding conformation and pulling pathways, we speculate that some conformations with stronger binding may be lost in collecting the smallest dataset C. This provides strong evidence to support the suggestion that the peptide undergoes significant conformational changes upon adsorption on a surface as Tang et al. proposed^4^. However, it is very interesting to see from Fig. 3a that all four curves converge around 17 *k*_B_*T*. As stated in the introduction section, the adsorption free energy is dominated by the small work values, and what really matters is the overlap of the distribution in the small work region.

Fourth, deletion of the GGG linker in data set D shows no impact on the estimated adsorption free energy. One reason the GGG linker has little impact is because the glycine residues have no functional side group to interact with the gold surface. Additionally, the strong hydrophilicity of the cysteine carboxylate, as well as the carbonyl groups of the maleimide and amide groups in the functionalized PEG linker, pulls it away from the surface and prevent their interaction with the gold substrate. Additional details regarding the impact of the linker are provided in Supplementary Note 2. Computational experiments were performed on the simulated molecule (SM) shown in Supplementary Fig. 4 and the results are shown in Supplementary Fig. 5.

Last, we can compare our estimates with analytical solutions. The contour lines obtained from Eq. (5) in Fig. 3b show that a particular free energy value can be obtained by infinite combinations of the shape and rate parameters *α* and *λ*. Our estimates coincide with the contour curve of free energy ~17 *k*_B_*T*.

Based on the above discussion, we conclude that the adsorption free energy of A3 peptide on gold surface is 16.77 ± 0.07 *k*_B_*T*.

### Free adsorption energy from MP-SPR experiments

To compare the above result with macroscopic measurements, we have collected a range of kinetic data from MP-SPR experiments at 0.1, 0.5, 1, and 25 μM peptide concentrations. They are fitted by least-squares regression to Langmuir isotherms given by^3^:

7 $$\theta \left( t \right) = \frac{C}{{C + K_{\mathrm{D}}}}\left( {1 - e^{ - k_{{\mathrm{obs}}}t}} \right)$$

where *θ*(*t*) is the fraction of surface coverage, *C* is the concentration, *k*_obs_ = *k*_a_*C* + *k*_d_ is the observed rate constant where *k*_a_ and *k*_d_ are the association and dissociation rate constants, *K*_D_ = *k*_d_/*k*_a_ is the equilibrium dissociation constant, *θ*_eq_ = *C*/(*C* + *K*_D_) is the equilibrium fraction of surface coverage, and *t* is time.

**Fig. 4:**
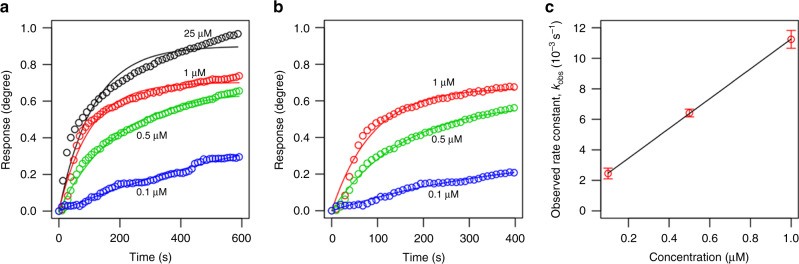
Fitting of the multi-parametric surface plasmon resonance (MP-SPR) experimental data using the Langmuir model. **a** Kinetic profiles obtained by MP-SPR at four different concentrations, fitted in the full range 600 s. The experimental data is denoted by the circles and the solid lines are the respective fitting curves. **b** The initial 400 s data at low concentrations used to extract kinetic rate constants. **c** Observed rate constants (*k*_obs_) plotted as a function of the peptide concentration. Linear regression was used to calculate the adsorption (*k*_a_) and desorption (*k*_d_) rates.

One of the key assumptions for the Langmuir model is that peptide-peptide interactions are ignored. This implies that the Langmuir model should work well in infinite dilute solutions. As Fig. 4a, b shows, at the initial short time period up to 400 s and under low concentrations (0.1, 0.5, and 1 μM), the Langmuir model fits very well with the kinetic profiles obtained by MP-SPR. However, at high concentration 25 μM, obvious deviation of the experimental data from the fitting curve can be observed. Therefore, we used the kinetic profiles obtained at the three lowest concentrations to extract the kinetic rate constants. We repeated the analysis using three different time periods 300 s, 350 s, and 400 s to make sure that overfitting has not occurred.

For each concentration, the fitting analysis shown in Fig. 4b produces a pair of values *θ*_eq_ and *k*_obs_. We have used linear regression to calculate the adsorption (*k*_a_) and desorption (*k*_d_) rate constants as shown in Fig. 4c. The obtained values are listed in Table 2.

**Table 2:** Kinetics extracted from MP-SPR data.

Duration (s)	*k*_a_ (M^−1^ s^−1^)	*k*_d_ × 10^−3^ (s^−1^)	*K*_D_ × 10^−7^ (M)	Δ*G*_ads_ (*k*_B_*T*)
300	10568.27 ± 1008.29	1.43 ± 0.65	1.36 ± 0.62	−15.81 ± 0.46
350	9882.01 ± 244.17	1.71 ± 0.16	1.73 ± 0.16	−15.57 ± 0.09
400	9769.10 ± 69.66	1.49 ± 0.05	1.53 ± 0.05	−15.69 ± 0.03
Average	10073.13 ± 432.50	1.55 ± 0.15	1.54 ± 0.19	−15.69 ± 0.13

Adsorption rate (*k*_a_), desorption rate (*k*_d_), equilibrium dissociation constant (*K*_D_), and free adsorption energy estimated for three different initial time periods.

According to the fitting, the average value obtained for *K*_D_ is 0.154 ± 0.019 µM. Using equation ${\mathrm{{\Delta} }}G = RT\ln \frac{{K_{\mathrm{D}}}}{{c^ \ominus }}$, where *R* is the ideal gas constant, *T* is the temperature in Kelvin and *c*^⊖^ is the standard reference concentration (1 M), the adsorption free energy Δ*G* for this system is estimated as −15.69 ± 0.13 *k*_B_*T*, in excellent agreement with the result obtained from single-peptide pulling experiments.

### Molecular dynamics simulation

**Fig. 5:**
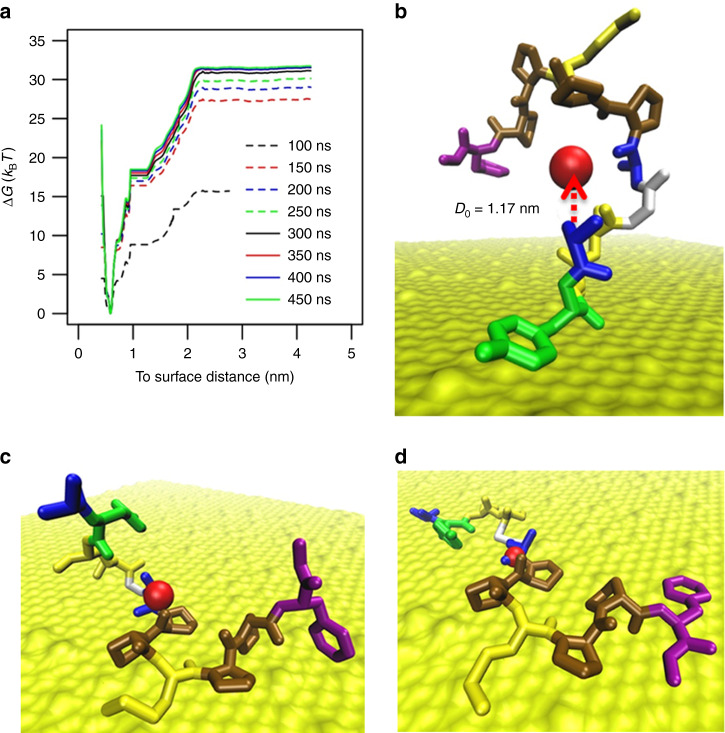
Molecular dynamics simulation. **a** The calculated potential of mean force (PMF) using adaptive biasing force method. After 400 ns simulation, the solution converges. **b** Snapshot of peptide conformation in the window centered at 1.5 nm; when the distance of the mass center of all the alpha carbons of the peptide from surface is ~1.2 nm, the N-terminal touches the surface and stays for a while, which corresponds to the plateau around 1 nm shown in (**a**). **c** Snapshot of peptide conformation in the window centered at 0.9 nm. **d** Snapshot of peptide conformation in the window centered at 0.3 nm. The center-of-mass is represented by a red sphere.

We have further validated the experimentally determined adsorption free energy using the adaptive biasing force method (ABF)^33,35,36^. First, we constructed the potential mean force (PMF) profile as described in the methods section. The reaction coordinate *D* is defined as the distance from the mass center of the peptide alpha carbons to surface. The PMF profile in Fig. 5a shows four different phases: (1) when *D* > 2 nm, the peptide is in the bulk solvent; (2) when 2 > *D* > 1.2 nm, biased diffusion occurs to the peptide; (3) when 1.2 > *D* > 0.9 nm, the N terminal of the peptide touches the surface, which is shown in Fig. 5b. This yields a narrow plateau around 1 nm in the PMF curve; (4) When 0.9 > *D* > 0.5 nm, the peptide experiences conformational changes and adsorbs on the surface. Representative peptide conformations are shown in Fig. 5c, d. This adsorption scenario is in agreement with the adsorption process observed by other groups^37^.

The above observations allow us to set a separation distance *D*_0_ at any position in the plateau 1.2 > *D* > 0.9 nm to define an adsorbed state (*a*) for distances shorter than *D*_0_ and a solution state (*s*) for larger distances than *D*_0_. Thus, the average probability of finding the peptide in the adsorbed and solution states is proportional to *ρ*_a_ and *ρ*_*s*_, which can be calculated by^38–40^

8 $$\rho _{\mathrm{a}} = \frac{1}{{D_0 - D_{{\mathrm{min}}}}}\mathop {\int}\limits_{D_{{\mathrm{min}}}}^{D_0} {e^{ - \beta {\Delta} G\left( z \right)}} dz$$

9 $$\rho _{\mathrm{s}} = \frac{1}{{D_{{\mathrm{max}}} - D_0}}\mathop {\int}\limits_{D_0}^{D_{{\mathrm{max}}}} {e^{ - \beta {\Delta} G\left( z \right)}} dz$$

where *D*_min_ = 0.5 nm, *D*_0_ = 1.0 nm, and *D*_max_ = 4.2 nm.

Next, we have calculated the adsorption free energy from PMF data collected at 350 ns, 400 ns, and 450 ns using the following equation

10 $${\Delta} G_{{\mathrm{ads}}} = - k_{\mathrm{B}}T\ln \left( {\frac{{\rho _{\mathrm{a}}}}{{\rho _{\mathrm{s}}}}} \right)$$

The averaged adsorption free energy ${\Delta} G_{{\mathrm{ads}}} = - 17.598 \pm 0.265\,k_{\mathrm{B}}T$ is in good agreement with experimental values.

We have noticed that the estimated adsorption free energy using Eq. (10) depends on the choice of the separation distance *D*_0_. If we choose *D*_0_ around 2 nm counting the biased diffusion peptide as a bound peptide, the estimated adsorption free energy is about 26.82 *k*_B_*T*, which gives the upper bound of the adsorption free energy. Between the two adsorption free energy values 12.8 and 106 *k*_B_*T* reported previously^4,20^, our result supports the hypothesis that significant conformational change of A3 upon adsorption on gold surfaces contributes to the entropic change.

In the SMPE measurement, a linker –GGGC-MAL-(PEG)_n_– is covalently attached to the C-terminus of the peptide A3. In the MP-SPR measurement and ABF-MD simulation, only the peptide A3 is investigated. But comparable adsorption free energy has been obtained. Is the agreement a coincidence? To answer this question, we have performed two additional experiments. The first one is to vary the linker by deleting GGG in the data set D. The second one is to apply the same approach to a new peptide-graphene system as described in Supplementary Note 3 and displayed in Supplementary Fig. 6. In both cases, we have seen little impact of the linker on the estimated adsorption free energy. One explanation is that in choosing the right bound state A in the data process, the impact of the linker is effectively excluded. Indeed, we have seen the results depend on the choice of the bound state A by varying the parameter *mulBase*, as shown in Fig. 1c. The other explanation is that the maleimide-based linker is away from the surface. We have discussed this possibility in Supplementary Note 2. In conclusion, the approach proposed here is robust in extracting adsorption free energies from surface bound peptides.

## Discussion

The Jarzynski equality has been widely used to evaluate equilibrium free energy differences from single-molecule pulling experiments. We have estimated the adsorption free energy of the A3 peptide on a gold surface using different estimators of the Jarzynski equality. The finite sample average estimator is biased by small work values. Based on small experimental sample sizes, those rare trajectories having small work values may be collected or may be lost, so divergent results may be obtained so that extreme caution must be exercised in estimating the free energy using the average JE estimator. The hard criterion is that the same free energy should be obtained using different pulling rates. When the Gaussian work distribution approximation has been proven to be in the regime of near-equilibrium, where the work fluctuation is about *k*_B_*T*, we have demonstrated that the Gaussian work distribution estimator gives nonphysical results when the work fluctuation is much greater than *k*_B_*T*. Here we have presented the first real-world example demonstrating a gamma work distribution with SPME. Using a gamma distribution estimator, four datasets produced consistent results comparable to those obtained from MP-SPR and molecular dynamics. Our results demonstrate the advantage of the gamma distribution estimator in estimating the adsorption free energy of peptide adsorbed on surfaces. However, our studies have focused on peptide-material surface interactions like gold and graphene, and further studies are needed using other biological and multivalent interactions to fully understand the application of statistical gamma work distribution. Nonetheless, our results show that the appropriate designation of the statistical family of the work distribution is critical in applying JE in biological systems.

## Methods

### Chemicals and materials

Bruker MLCT AFM tips modified with (3-Aminopropyl)triethoxysilane (APTES) were purchased from Novascan Technologies, Ames, IA USA. Heterobifunctional PEGs, MAL-PEG5000-SCM, MAL-PEG2000-SCM, and HO-PEG2000-NHS, were purchased from Creative PEGWorks. The gold-binding A3 peptide was purchased with a –GGGC terminal group for covalent linkage to the maleimide group on the PEG linker. The peptide AYSSGAPPMPPF-GGGC was purchased from GenScript at 99.6% purity. The peptide AYSSGAPPMPPF-C was purchased from New England Peptide at >95% purity. Au (111) thin films on freshly cleaved mica substrates were purchased from Phasis Sàrl, Geneva – Switzerland. Chloroform, 99%, anhydrous was purchased from Sigma Aldrich. Triethylamine, ≥99%, was purchased from Sigma Aldrich and stored over 4 Å sieves.

### AFM Measurements

Force spectroscopy experiments were performed on a Bruker Dimension Icon AFM with NanoScope V Controller under aqueous conditions. Deflection sensitivity calibration was performed on a sapphire substrate and the thermal noise method was used to estimate the cantilever spring constant. A maximum force of 500 pN was set during contact with the sample before tip retraction. A 1–2 s dwell time was set to allow time for peptide interaction with the surface.

### AFM Tip preparation

All glassware for PEG modifications was baked for at least 1 h prior to use at 100 °C under vacuum. PEG solutions were prepared in anhydrous chloroform at 5 mM concentration. Modification strategies differed for the different data sets presented. For the AFM tips used to collect data sets A, C, and D, two different PEGs were used to control the density of the A3 peptide on the tip. MAL-PEG5000-SCM was used as the linker to provide a parabolic signature in the AFM F-D curves with an unbinding separation distance around 50 nm, and HO-PEG2000-NHS was used as a spacer molecule to control the density of peptide. These PEGs were mixed in a ratio of 1:7, respectively, and the APTES modified AFM tips were incubated in the solution for 1 h at room temperature. For data set B, the AFM tip was modified using MAL-PEG2000-SCM as the linker in the same concentration as the linker used in the other data sets, but with no spacer. The PEG modified tips were then washed three times with chloroform, dried briefly, and transferred into the peptide solution. The peptide solution was prepared by dissolving A3-GGGC peptide (for data sets A, B, and C) or A3-C peptide (for data set D) at a concentration of 0.2 mg/mL in 0.1 M sodium phosphate buffer with 0.01 M EDTA at pH 7.2. The tips were transferred into this solution and incubated for 4 h at room temperature. Tips were then rinsed two times in buffer, once in water, and then transferred into water for storage at 4 °C.

### Gold surface preparation

Gold (111) substrates were rinsed with water, followed by isopropanol, and cleaned using UV-ozone treatment for 10 min prior to use (PSD Pro Series UV-Ozone cleaner from Novascan Technologies).

### Data processing

Five steps were performed to detect a retraction force-distance curve (FDC) with a single-peptide binding event. First, the rupture point was detected by calculating the derivative of FDC. The derivatives at each point *i* of FDC were calculated in a window [*i* − *r*, *i* + *r*] using the linear regression fitting method, where r is the radius of the window. The radius r was increased step by step until the number of significant maxima in the derivative FDC stopped varying. Those significant maxima were corresponding to discontinuous points in FDC. The one far away from the surface is the rupture point.

Second, the force in the segment of FDC away from the last rupture point was set as a zero-force reference within the fluctuation limit. The linear regression fitting method was used to align the mean force to zero. The standard deviation *σ* of the force was estimated.

Third, the minima of FDC were detected using the convolution scheme^24^. The one before the rupture point with force value smaller than the noise level −*LOD* * *σ* was accepted as a binding site, where *LOD* is a parameter given by a user. Fourth, a zero force point was detected before the binding site in the baseline allowing *mulBase* * *σ* fluctuation.

Last, the stretching curve between the zero force point and the binding site was fitted by the worm-like chain (WLC) model^41^:

11 $$F\left( \gamma \right) = \frac{{k_{\mathrm{B}}T}}{{l_{\mathrm{p}}}}\left[ {\frac{1}{4}\left( {1 - \frac{\gamma }{L}} \right)^{ - 2} + \frac{\gamma }{L} - \frac{1}{4}} \right]$$

where *F* is the force, *γ* is the polymer extension, *l*_p_ is the persistence length of ~0.37 nm, *L* is the contour length of the polymer, *k*_B_ is the Boltzmann constant and *T* the temperature. The work disribution was fitted using the R package fitdistrplus^42^.

### Multiparametric surface plasmon resonance (MP-SPR)

MP-SPR studies were conducted using a BioNavis SPR Navi 200 Spectrometer, BioNavis, Finland and gold sensors (BioNavis) (111) single crystalline surfaces. The peptide A3 was synthesized in-house with 95% purity. Polypeptide solutions (0.1, 0.5, 1, and 25 µM) were prepared in type 1 water at pH 7. The cell temperature and flow rate were 20 °C and 23 µL min^−1^, respectively. Each experiment had a total duration of half an hour. The region of binding was extracted from the data and fitted using the Langmuir isotherm to obtain the dissociation constant (*K*_D_) and adsorption free energy (Δ*G*).

### Molecular dynamics simulation

**Fig. 6:**
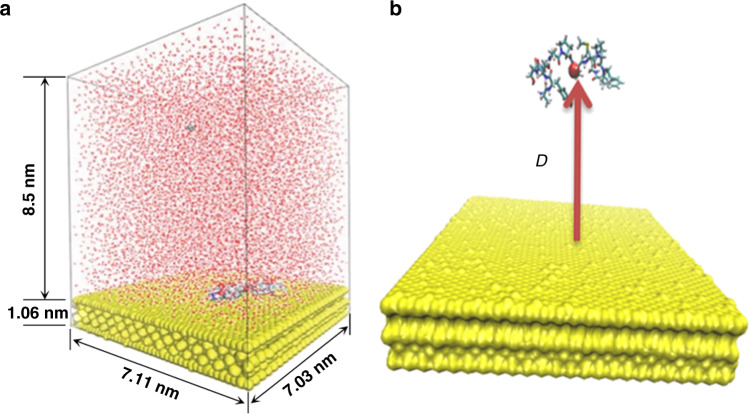
The simulated system. **a** Snapshot of the simulated system consisting of water, peptide, one chloride ion and surface; **b** the distance of the mass center of all alpha carbons in the peptide from a gold surface is chosen as the collective variable in PMF calculation.

The initial structure of the peptide A3 was predicted using I-TASSER program^43^. An Au(111) surface of size 7.105 nm × 7.032nm with additional interaction sites accounting for polarizable effect was kindly provided by Bellucci^44^. Since the contour length of a 12-mer peptide A3 is about 4.2 nm, a 8.5 nm high water box consisting of 14064 water molecules was put on the gold surface to avoid image interaction of the peptide with the bottom surface of the gold slab when periodic boundary condition was imposed. The peptide A3 was randomly immersed in the water. The model was assembled using VMD 1.9.2 program^45^. A snapshot of the modeling system is shown in Fig. 6a.

Molecular dynamics simulations were carried out using the NAMD 2.10 software package^46^ and the polarisable Go1P-CHARMM force field^47,48^ with a time step of 1 fs and periodic boundary conditions in all three directions. The system was first minimized using a conjugate gradient and line search algorithm. Then the system was incrementally heated from 0 to 300 K in steps of 30 K every 1000 fs. The system was then allowed to equilibrate at constant temperature and pressure (300 K, 1 atm, NPT ensemble) for 10 ns. The particle mesh Ewald (PME) method^49^ was used to calculate the long-range electrostatic interactions. The cut-off, switching, and pair-list distances were chosen as 1.0 nm, 0.9 nm, and 1.2 nm, respectively. The Langevin piston Nose-Hoover method^50,51^ was used to control the pressure with piston period 200 fs and damping time scale 100 fs. The temperature was controlled through Langevin dynamics with damping coefficient 5/ps.

### Calculation of potential of mean force

To compute the potential of mean force (PMF) using the ABF algorithm, the distance *D* of the mass center of all alpha carbons in A3 from the gold surface were chosen as the collective variable, as shown in Fig. 6b. To enhance the sampling, the region of interest *D* ∈ (0, 42) was divided into 7 equally spaced windows of width 6 Å. The window centers were at 3, 9, 15, 21, 27, 33, and 39 Å, respectively. To obtain the initial configuration at each window, seven constraint molecular dynamics simulations were run to bring the peptide to each window center by imposing a harmonic force on the collective variable *D*. To obtain smooth free energy profile, each window was divided into 60 bins so that the bin size *δD*_*Y*_ was 0.1 Å. Therefore, the free energy difference was reconstructed by^33,36^

12 $${\Delta} A = A(D_Y^b) - A(D_Y^a) = \mathop {\int}\limits_{D_Y^a}^{D_Y^b} {\frac{{dA}}{{dD_Y}}} dD_Y = - \delta D_Y\mathop {\sum}\limits_{i = 1}^{420} {\left\langle {F_{D_Y}} \right\rangle _i}$$

where $F_{D_Y}$ is an instance force acting on the reaction coordinate in the *i*th bin.

To implement Eq. (12), we have first positioned the peptide inside each window using a biased harmonic potential with an actual force constant 30 kcal/mol/Å^2^. Then half-harmonic potentials imposed at the boundary of each window with sufficiently strong harmonic restraints 100 kcal/mol/Å^2^ were used to constrain the mass center of the peptide inside each window. ABF simulations were conducted for 450 ns in each window so that the total samples were 450,000,000 per window and the total simulation time was 3150 ns for the whole system.

## Supplementary information

Supplementary Information

## Data Availability

The datasets analyzed in the study are available in Github https://github.com/comtook/afmFreeEnergy.

## Code availability

The codes generated and used in the study are available in Github https://github.com/comtook/afmFreeEnergy.
