# Supplementary material for: Gamma estimator of Jarzynski equality for recovering binding energies from noisy dynamic data sets
Source: Nat Commun. 2020 Nov 2;11:5517. doi: 10.1038/s41467-020-19233-7 (PMC7606380; doi:10.1038/s41467-020-19233-7)
Supplement: Supplementary file 1 — Supplementary Information [file 41467_2020_19233_MOESM1_ESM.pdf]

Supplementary Information

Gamma Estimator of Jarzynski Equality for Recovering Binding Energies from Noisy Dynamic Data Sets

Zhifeng Kuang[1], Kristi M. Singh[1], Daniel J. Oliver[2], Patrick B. Dennis[1], Carole C. Perry[2] and Rajesh R. Naik[1]*

[1]Air Force Research Laboratory, Wright-Patterson Air Force Base, Dayton, OH 45433, USA

[2]Biomolecular and Materials Interface Research Group, Interdisciplinary Biomedical Research Centre, School of Science and Technology, Nottingham Trent University, Clifton Lane, Nottingham NG11 8NS, UK

*E-mail: Rajesh.Naik@us.af.mil

Supplementary Figures

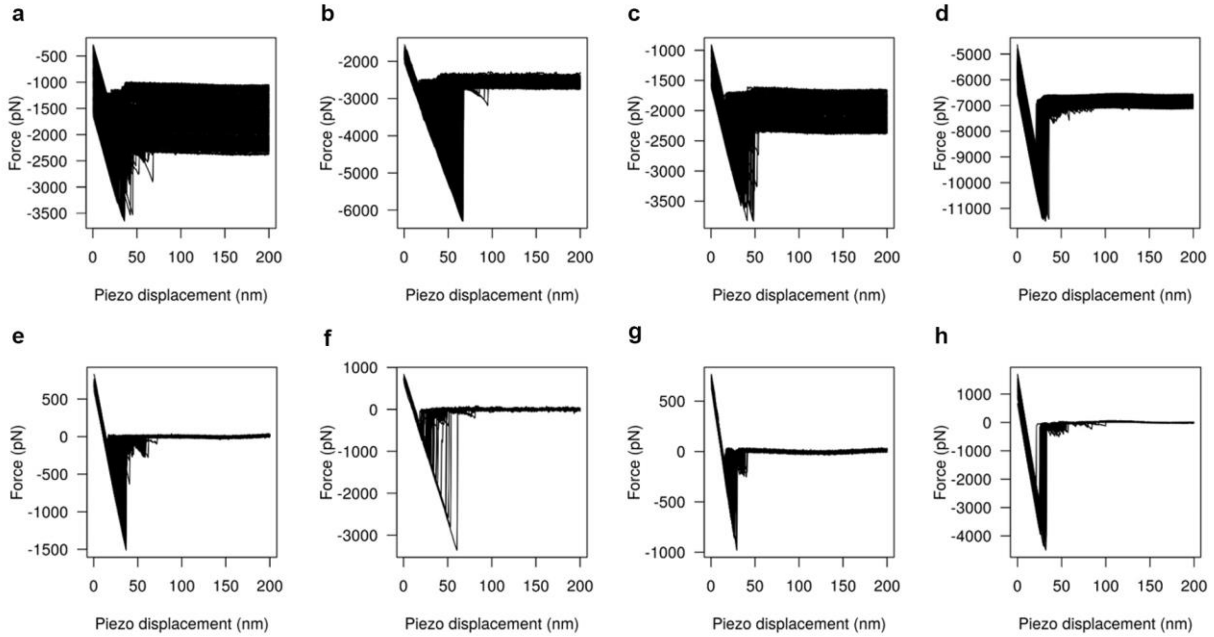

Supplementary Figure 1. The experimental data. a-d Four sets of raw data collected from single-molecule pulling experiments. **e-h** The selected data based on the parameters listed in Table 1 in the main text.

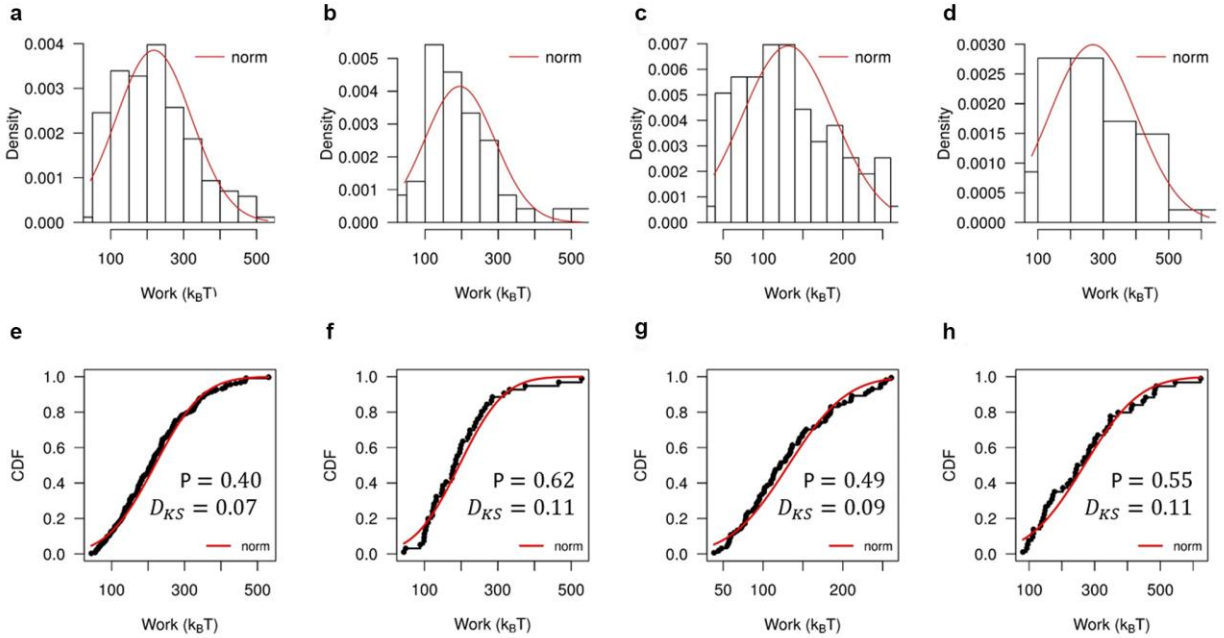

Supplementary Figure 2. The statistics of Gaussian fitting. a-d Histograms of work values fitted with Gaussian distribution for dataset A, B, C and D, respectively. **e-h** The empirical cumulative distribution function (CDF) comparing with theoretical Gaussian distribution for datasets A, B, C and D, respectively. $\boldsymbol{D_{KS}}$ is the Kolmogorov-Smirnov test-statistic. $\boldsymbol{P}$ is the P-value representing the likelihood of observing the test-statistic.

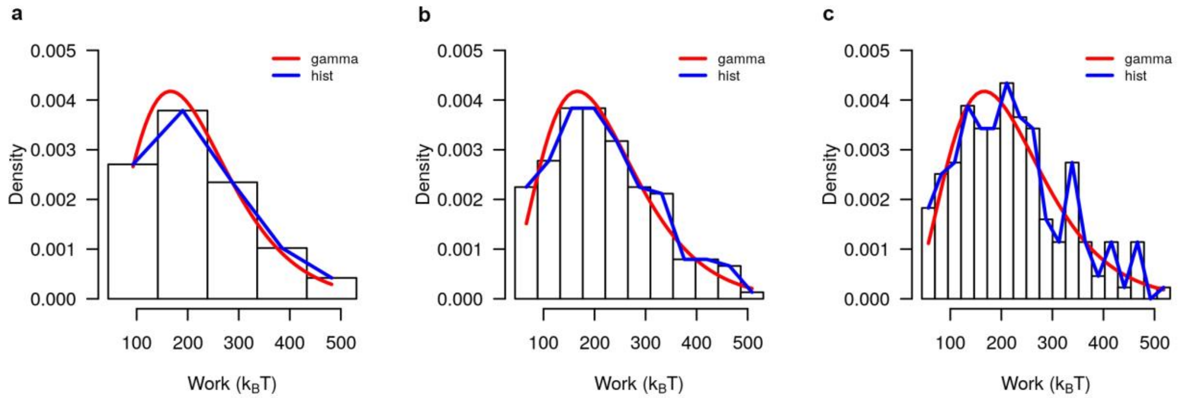

Supplementary Figure 3. Effect of bin sizes on estimated density function from histogram. The red curve is estimated using maximum likelihood method. **a** 5 bins; **b** 11 bins; **c** 19 bins.

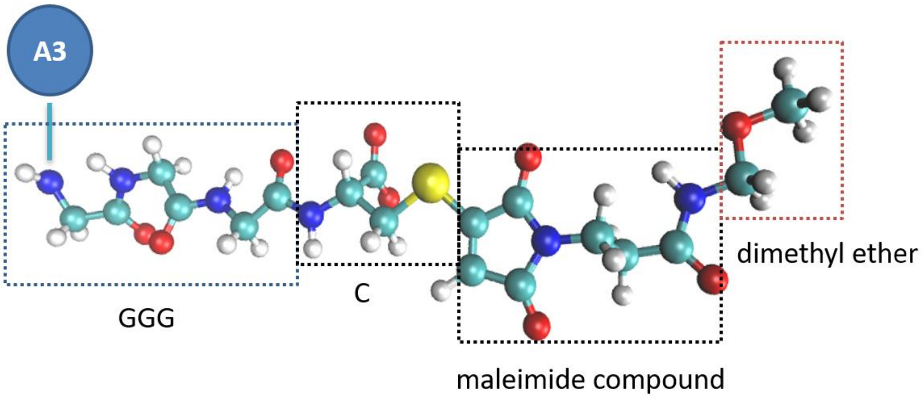

Supplementary Figure 4. The structure of the simulated molecule for investigation of -GGGC- impact on adsorption. For clarity, the peptide A3 is represented by a sphere.

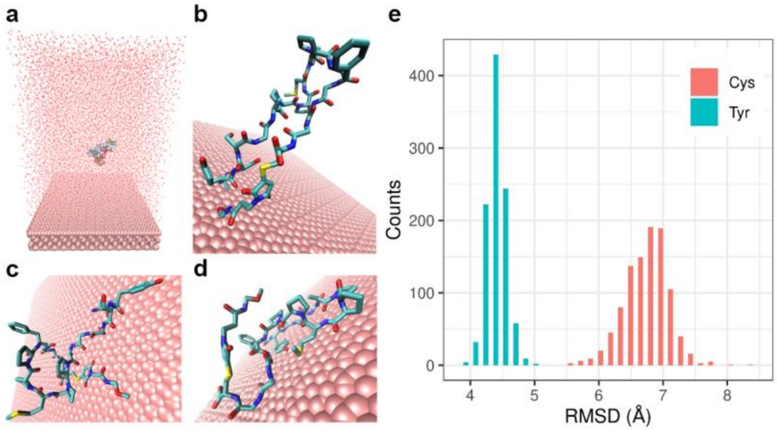

Supplementary Figure 5. Computational experiments to test the effect of the linker. **a** the simulated system. **b** the end-point conformation after first pulling experiment. **c** the end-point conformation after second pulling experiment. **d** the most likely adsorbed conformation. **e** the histogram of distance of alpha carbons to surface in tyrosine and cysteine.

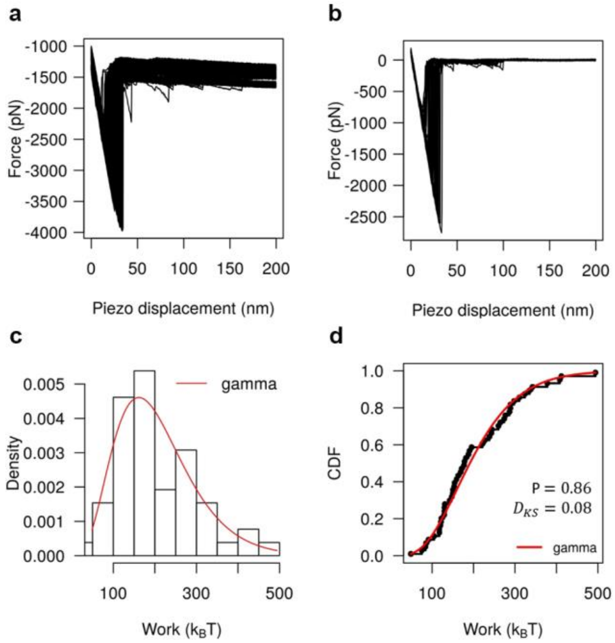

Supplementary Figure 6. Peptide P1 interaction with graphene sheets. a the retraction trajectories acquired from single-molecule pulling experiments. **b** the selected trajectories for free energy calculation. **c** the work distribution fitted with gamma distribution. **d** the empirical cumulative distribution function comparing to theoretical gamma CDF shows the fitting is good.

Supplementary Tables

Supplementary Table 1. Experimental parameters extracted from measurement records. Velocity = displacement/duration; Deviation=mean standard deviation of force in bulk region.

	Retract Timestep (s)	Retract duration (s)	Piezo displacement (nm)	Velocity (nm/s)	Temperature (K)	Spring constant (pN/nm)	Bulk force deviation (pN)
A	0.000775	0.3960185	199.6094	504.0423	294.15	65.0158	9.04 ± 0.41
B	0.000575	0.2938281	199.6094	679.3407	294.15	66.1654	14.19 ± 5.59
C	0.000775	0.3960185	199.6094	504.0423	294.15	65.0158	11.03 ± 1.10
D	0.000768	0.3924511	199.6093	508.6225	294.15	239.179	19.72 ± 7.58

Supplementary Notes

Supplementary Note 1: Limitations in deriving a distribution model from histogram

Assuming a parametric distribution model such as norm and gamma, the maximum likelihood method is consistent and efficient in estimating the parameters which determine the density function. However, if a particular parametric distribution family is not known, a simple way to nonparametrically learn a distribution model directly from the data is to make a histogram. A histogram gives us a piecewise constant estimate of the density function since the probability density within each bin is a constant. It has been proved that the asymptotic integrated mean squared error between a true density function $f(x)$ and a piecewise density function from histogram is[1]

$$\boldsymbol{IMSE} = \frac{\mathbf{1}}{\boldsymbol{nh}} + \frac{\boldsymbol{h}^{\mathbf{2}}}{\mathbf{12}} \int \left(\boldsymbol{f}'(\boldsymbol{x})\right)^{\mathbf{2}} \boldsymbol{dx} \qquad \mathbf{1}$$

where n is the number of data points, h is the bin size, $f'(x)$ is the first derivative of the true density function. Differentiating $IMSE$ with respect to h and setting it equal to zero, we obtain the optimal h_{opt} as

$$\boldsymbol{h_{opt}} = \left(\frac{\mathbf{6}}{\boldsymbol{n} \int \left(\boldsymbol{f}'(\boldsymbol{x})\right)^{\mathbf{2}} \boldsymbol{dx}}\right)^{\mathbf{1/3}} \qquad \mathbf{2}$$

Unfortunately, $f'(x)$ is unknown. But Supplementary Equation 2 gives some rules to choose the bin size. If the true density changes rapidly, we need to choose narrow bins. If the density is relatively flat, we should choose wide bins. The bin size should be proportional to $n^{-1/3}$. Setting

the optimal bin size is a challenging research topic in nonparametric statistics. The R program offers four options to set the bin size. In generating the histograms in Figure 2, Supplementary Figure 2 and Supplementary Figure 6, we have used the default option. Supplementary Figure 3 shows the dependence on bin size of the estimated density function from a histogram using data set A. Taking the gamma density function estimated from maximum likelihood method as the true one and comparing the density values at the middle points of each bin, the root mean square error between the true density and estimated one is 0.00014, 0.00036, and 0.00058 for 5, 11 and 19 bins, respectively. The larger bin size yields better fit into the true one, as shown in Supplementary Figure 3a.

Supplementary Note 2: Impact of the linker

In the single molecule pulling experiment, a GGGC linker is included to provide freedom of rotation and to facilitate linkage to the maleimide group on the PEG linker. Additional experimental data (set D in main text) after deleting GGG yields the same adsorption free energy. In MP-SPR measurements, only A3 peptide is studied and yet comparable adsorption free energy is obtained. These findings indicate that the linker has minimal impact on peptide binding. To understand the mechanism leading to the agreement with and without GGG, molecular dynamics simulations have been carried out to observe how the molecule adsorbs on surface.

Since cysteine is included for conjugation of the peptide via the maleimide-thiol interaction, we must include the maleimide compound in our simulation. Supplementary Figure 4 shows the simulated molecule (SM) composed of peptide A3-GGGC-Maleimide compound. The maleimide compound is terminated with a dimethyl ether group to take use

of existing CHARMM PEG monomer force field parameters. The topology and parameters for the maleimide compound compatible with the CHARMM force field are provided by SwissParam.[2]

First, the SM was equilibrated in a water box of $7.105 \times 7.032 \times 10.632\, nm^3$ dimension for 100 ns at constant temperature 300 K and pressure 1 atm. Second, the equilibrated box was put right above Au(111) surface of the same xy dimension as shown in Supplementary Figure 5a. The whole system was equilibrated for 100 ns at constant temperature 300 K. Third, steered molecular dynamics simulations were carried out to adsorb the SM on the surface in a reasonable simulation time. Choosing the carbon in the methyl group at the end as the tagged atom, the SM was pulled towards the surface at a constant rate of 2×10^9 nm/s for 2 ns in water (corresponding to the approach phase in AFM experiment). It was found that the tyrosine residue initiates binding and the SM stands on the surface as shown in Supplementary Figure 5b. Since we know the A3 peptide is able to bind to the surface, we have repeated the pulling process by changing the centre of mass of the first nine alpha carbons as the tagged dummy atom at the A3 end. After the second steered molecular dynamics simulation, most of the SM is on the surface as shown in Supplementary Figure 5c. Finally, to obtain the adsorbed conformation corresponding to minimum energy, temperature replica exchange molecular dynamics simulations[3] of 64 replicas were performed with temperatures ranging from 298.15 K to 498.15 K for a total of 1.92 microsecond simulations. The conformation of lowest potential energy was further equilibrated at constant temperature 300 K and pressure 1 atm for 100 ns. The last 10 ns trajectories were used to get the coordinates of alpha carbons of the cysteine at one side and tyrosine at the other side. The end-point conformation is shown in Supplementary Figure

5d. It is interesting to see that the maleimide-based linker is not adsorbed on the gold surface, which is in agreement with experimental observation from Lee et al. which shows that even at low density the maleimide groups on a modified gold nanoparticle are still accessible.[4] The histograms of the distance, for the alpha carbons of the cysteine and tyrosine, from the surface are shown in Supplementary Figure 5e. From the distance distribution in Supplementary Figure 5e and conformation in Supplementary Figure 5d, it is clearly seen that the tyrosine is adsorbed on the surface, whereas cysteine is further away from the surface. Because of the strong hydrophilicity of the cysteine carboxylate and the carbonyl groups of the maleimide and amide groups, the linker -GGGC- is oriented away from the surface, so that it does not bind on the surface and has little contribute to the adsorption free energy of A3 on the gold surface.

Supplementary Note 3: Applicability to other systems

To test whether the approach is applicable to other model systems, we collected a set of data for peptide P1 (HSSYWYAFNNKT) interaction with a graphene surface.[5] The graphene-binding P1 peptide was purchased with a –GGGC terminal group to provide freedom of rotation and facilitate linkage to the maleimide group on the PEG linker.[6-8] The peptide, HSSYWYAFNNKT-GGGC, was purchased from GenScript at 98.9% purity. AFM tips were prepared from APTES modified DNP Bruker probes purchased from Novascan Technologies, Ames, IA USA. PEG modification was performed using the same protocol as data sets A, C, and D in the main text and peptide modification was performed following the same protocol as in the main text, using P1-GGGC as the peptide of interest. Data were collected on a Bruker Dimension Icon AFM with NanoScope V Controller operated in contact mode under aqueous conditions. Approach and retraction velocities were 200 nm/s with a 0 s surface delay and a calculated compression force of 287 pN prior to tip

retraction. The substrate consisted of 8 layers of graphene on a silicon wafer that was prepared according to the uniform graphene procedure noted in a previous publication.[9]

The retraction trajectories are shown in Supplementary Figure 6a. The selected trajectories for free energy estimates are shown in Supplementary Figure 6b. Supplementary Figure 6c shows the gamma density function using the shape and rate parameter $\alpha = 4.635,\ \lambda = 0.0225$ which maximize the likelihood function. The P-value for the fitting is 0.86. Using the equation $\Delta G_{GA} = \alpha ln\left(\frac{\lambda+1}{\lambda}\right)$, we have estimated the adsorption free energy as $17.68\ k_B T$. Using SPR, we have also measured the dissociation constant $K_D = 4.1 \times 10^{-8} M$, which can be converted to the adsorption free energy $17.01\ k_B T$ using equation $\Delta G = RT \ln \frac{K_D}{c^{\ominus}}$, where R is the ideal gas constant, T is the temperature in Kelvin and $c^{\ominus}$ is the standard reference concentration (1 M). The excellent agreement shows promise in applying the approach to other peptide-surface interaction systems.

Supplementary References

1. Hollander, M., Wolfe, D. A., & Chicken, E. *Nonparametric Statistical Methods, Third Edition* (John Wiley & Sons, Inc., Hoboken, New Jersey, 2014).
2. Zoete, V., Cuendet, M. A., Grosdidier, A., & Michielin, O. SwissParam: a fast force field generation tool for small organic molecules. *J. Comput. Chem.* **32,** 2359-2368 (2011).
3. Sugita Y., & Okamoto Y. Replica-exchange molecular dynamics method for protein folding. *Chem. Phys. Lett.* **314,** 141-151 (1999).
4. Lee, J. C. et al. Exploring maleimide-based nanoparticle surface engineering to control cellular interactions. *ACS Appl. Nano Mater.* **3,** 2421-2429 (2020).
5. Kim, S. N. et al. Preferential binding of peptides to graphene edges and planes. *J. Am. Chem. Soc.* **133,** 14480-14483 (2011).
6. Matin, T. R. et al. Single-molecule peptide-lipid affinity assay reveals interplay between solution structure and partitioning. *Langmuir* **33,** 4057-4065 (2017).
7. Kim, S. O. et al. Correlating single –molecule and ensemble-average measurements of peptide adsorption onto different inorganic materials. *Phys. Chem. Chem. Phys.* **18,** 14454-14459 (2016).
8. Kim, B. H. et al. Single-molecule force microscopy force spectroscopy study of Aβ-40 interactions. *Biochemistry* **50,** 5154-5162 (2011).
9. Kim, S. S. et al. Biotic−abiotic interactions: factors that influence peptide−graphene interactions. *ACS Appl. Mater. Interfaces* **7,** 20447-20453 (2015).
